# Indents

**Published:** 1919-02

**Authors:** 


### INDENTS

☞Brief Articles of Technical or Scientific Value will receive notice and comment. Contributions invited.

---

The Work of the Rockefeller Foundation. George E. Vincent, president of the Rockefeller Foundation, outlined recently the plans of the foundation which included an expedition to Central and South America headed by Major-General William C. Gorgas, formerly Surgeon-General, U. S. Army, to conquer yellow fever which now springs from a few small areas in Central and South America; the operation of a medical university in Peking, China, which is under construction with a cost of $6,000,000, and will be open in 1920, and which will contain eighteen university buildings, forty faculty residences, and a hospital with 200 beds, and a medical school soon to be begun at Shanghai. Subsidies are to be granted to existing missionary hospitals which will be standardized and offer internships for the university. Clinical stations and sub-hospitals will be established over China. For this work a total expenditure of $10,000,000 is expected, with an additional $250,000 to $500,000 annually for support.—*Journal A. M. A.* for Dec. 28.

---

A Method of Securing a Full Denture in Position. This method is particularly valuable in edentulous patients with a flat palate without ridges and without any points of retention along the alveolar border. Plaster impressions are necessary and vulcanization must be done on the model.

In the exact centre of gravity I trace on the model a circular groove whose circumference is about that of a shilling; the outer edge is left sharp, but the inner one is bevelled; the groove should be 1 mm. deep and 3 mm. wide. This area will be filled by a very thin layer of soft rubber having the same circumference as the groove, and covered by hard rubber to avoid subsequent breaking at this point of least resistance.

Vulcanization should be done very slowly and at a low temperature so as to preserve all the qualities of the soft rubber.

On the plate a relief is thus obtained and a small surface of soft rubber which makes perfect suction.

The disadvantages of rubber discs suctions are avoided by this method and all the advantages obtained.

This suction chamber does not injure the mucous membrane and permits the edentulous to learn to masticate rapidly.—Dr. Charbonneau in *L'Odontologie,* Aug., 1918.

---

THE NATURAL ARTICULATOR. The one dependable and truly anatomical articulator is the combination of the upper and lower jaws of the patient. Inasmuch as great variation of form of the bony structure of the human head maintains in the human race, the individual, as he presents himself to us for a service, furnishes us with our only true guide to the correct relation of that particular mandible and maxilla.

The articulator must, therefore, be only an instrument to maintain movement and relation in the absence of the patient.

The jaws of an edentulous person have no path or track upon which to functionate or to guide them in mastication, as is the case with those who have not lost their teeth. Therefore, while it is possible for any one to get accustomed to an inferior form of denture, and in time get some degree of service therefrom, our plain duty is to come as close to the natural state as we can by following nature. The results are just the difference between good results and results that are deficient. My experience has been that a greater degree of perfection is attained by following the methods employed by nature in the creation and development of the organs of mastication, and the way they are placed in the mouth of mankind.

One word regarding the so-called anatomical articulator, and where its real place is in prosthetic dentistry. I am strongly of the opinion that we have been trying to mystify ourselves in the past, and have been looking through the wrong end of the telescope in our effort to get at the facts.

As has been said, the bony structure, the articulating and functioning process of the maxilla and mandible, differ so widely in each individual case that it would be unscientific, to say the least, to try to place every one upon the same basis, inasmuch, as each patient brings with him his own truly anatomical articulator; why not make use of it to our own end? The only thing we demand of an articulator is that it maintains during the absence of the patient that which we have attained directly from the mouth of the individual.—Dr. Essig in *Journal N. D. A.*

---

Precautions on the part of Physicians to Avoid Influenza Infection. *To the Editor:*—I believe *The Journal* would do service by suggesting precaution that should be practiced by physicians and others in the sick room with influenza patients. Large numbers of Pennsylvania physicians are sickening, I am sure, because they are indifferent to danger and careless about protecting themselves from the spray of moisture from the mouth of the patient.

I am getting stories every day by the score of physicians examining the chests of influenza patients in the old careless fashion, leaning over the bed with the patient talking constantly and with no attempt to have the patient cover the cough or sneeze with a towel or handkerchief. The sick rate among physicians and the death rate are appalling, and I believe physicians are needlessly sacrificing themselves because of their failure to fully appreciate what they ought to do.

I have not had time to prepare an article and have only had time to glance over the series of splendid articles illustrated in *The Journal.*

Masks if properly used do offer a certain degree of protection apparently and yet the every-day precaution that we practice in handling the tuberculous should be practiced at all times by careful physicians.—B. Franklin Royer, M.D., Harrisburg, Pa., Acting Commissioner, Department of Health, Pennsylvania, in *Journal A. M. A.*

